# Clinical observational studies of potential participants’ current negative affect status for a clinical study of stem cell therapy for ischemic stroke: study protocol

**DOI:** 10.3389/fneur.2025.1429846

**Published:** 2025-04-10

**Authors:** Xixian Qin, Mengyao Li, Xuna Sun, Peipei Dong, Xiaofei Ji, Xiaoyan Li, Jing Liu

**Affiliations:** ^1^Emergency and Disaster Medical Center,Seventh Affiliated Hospital, Sun Yat-Sen University, Shenzhen, China; ^2^Stem Cell Clinical Research Center, The First Affiliated Hospital of Dalian Medical University, Dalian, Liaoning Province, China; ^3^Department of Neurology, First Affiliated Hospital of Dalian Medical University, Dalian, Liaoning Province, China; ^4^Dalian Innovation Institute of Stem Cell and Precision Medicine, Dalian, Liaoning Province, China

**Keywords:** patient participation, depression, anxiety, psychiatry, study protocol, mesenchymal stem cells, stroke, neurology

## Abstract

**Introduction:**

The number of clinical research projects on stem cell therapy for stroke has increased annually with the rapid development of stem cells and regenerative medicine technologies. Some evidence indicates that negative emotions can affect the recruitment, compliance, retention, satisfaction, and even treatment outcomes of participants in clinical research. However, knowledge is insufficient regarding patients’ negative emotions associated with their participation in potential stem cell clinical research studies. Therefore, the study aims to investigate the negative emotions and main influencing factors for potential participants in clinical research on stem cell therapy for stroke.

**Methods:**

This study protocol follows the Strengthening the Reporting of Observational Studies in Epidemiology Good Practice for Reporting Observational Studies. The questionnaire for this study will include 59 questions for potential participants regarding (1) their demographic characteristics, and (2) their levels of anxiety, depression, social support, general self-efficacy, and self-perceived burden.

**Discussion:**

This study’s main strength is that it will contribute evidence on key predictors of negative affectivity in potential participants undergoing clinical trials of stem cell therapy for stroke. The results will support stem cell clinical center researchers in intervening in potential participants’ negative emotions, which is important for clinical research managers and policymakers worldwide.

**Conclusion:**

The study protocol developed in this study was validated through a rigorous development process, demonstrating scientific validity for negative affect evaluation in stem cell clinical research. This instrument provides clinicians with a standardized assessment protocol to monitor treatment-emergent emotional distress during experimental interventions, showing particular promise in identifying early psychological risks associated with novel biological therapies.

## 1 Introduction

The *Annual Report on the Progress of Clinical Trials of New Drug Registration in China (2022)* released by the Drug Evaluation Centre of the State Food and Drug Administration (1), the Drug Clinical Trial Registration and Information Disclosure Platform recorded over 3,410 clinical trials in 2022. A record number of 46 new clinical trials of cell and gene therapy products were registered in 2022. The approval rate for clinical trials was 55.8%, while the efficiency of clinical trial initiation improved, with the overall proportion of participant recruitment initiated within 6 months reaching 91.5%. As of December 2023, the registered work of 141 stem cell clinical research institutions had been completed in China, and 127 stem cell clinical research projects had been finalized. Further, the number of clinical research participants has continued to increase.

For new treatment technologies, participants may experience varying degrees of negative emotions because they are concerned about the trial’s safety and effectiveness. Peng et al. (2) found that many patients with hemophilia who participated in clinical trials showed various physical and psychological symptoms, such as anxiety and depression. Additionally, participants lack an understanding of how they perceive stimuli and their coping strategies (3).

Negative emotions can affect treatment outcomes negatively. Some studies (4–8) have shown that negative emotions increase mortality and affect patients’ treatment outcomes, functional recovery, social activities, and quality of life. Negative emotions may cause participants to experience depression, cognitive decline, inattention, or distraction (9), which subsequently leads to reduced participant compliance and negative impacts on investment and cooperation in the treatment process. Clinical studies have shown that negative emotions increase the risk of adverse reactions (10). For example, participants with depressive symptoms may experience serious adverse events in clinical studies, such as suicide and self-harm (11, 12). Hence, negative emotions influence the participation of potential participants in clinical research. Moreover, negative affect may cause participants to be less interested in participating in clinical research, thereby reducing their participation, compliance, and retention. Chuangu et al. (13) found that anxiety and depression were independent risk factors for clinical trial participants withdrawing from studies. Therefore, it is particularly important to screen and intervene in response to negative emotions among potential participants in clinical research.

However, researchers typically do not adequately assess participants’ negative emotions in the clinical research process because the purpose and focus of stem cell clinical research is to examine the effects of treatment. Therefore, it is difficult for medical staff to detect the negative emotional status of stem cell clinical research participants or to implement timely and effective intervention measures.

As the ‘bio-psycho-social-environmental’ medical model becomes mainstream, the focus of clinical research should not be limited to the therapeutic effect. The participants’ negative psychological emotions must be seriously considered. The incidence of anxiety, depression, and negative emotions is high after a stroke (14) and thus negatively impacts the therapeutic effect (6, 8).

This project examines the negative emotional characteristics of potential participants for stem cell therapy in clinical research on stem cell therapy for stroke. The results of this study will provide a basis for the psychological care of this group during their formal participation in stem cell clinical research. Therefore, this study is intended to promote the screening and improve the mental health of this patient group and ensure the smooth progression of participants in stem cell clinical research.

### 1.1 Research purpose

This survey-based clinical observational study aims to use questionnaires to (1) evaluate the anxiety and depression status of participants in clinical research on stem cell therapy for stroke before receiving treatment and (2) analyze the influencing factors.

## 2 Materials and methods

### 2.1 Study design

**Figure 1:**
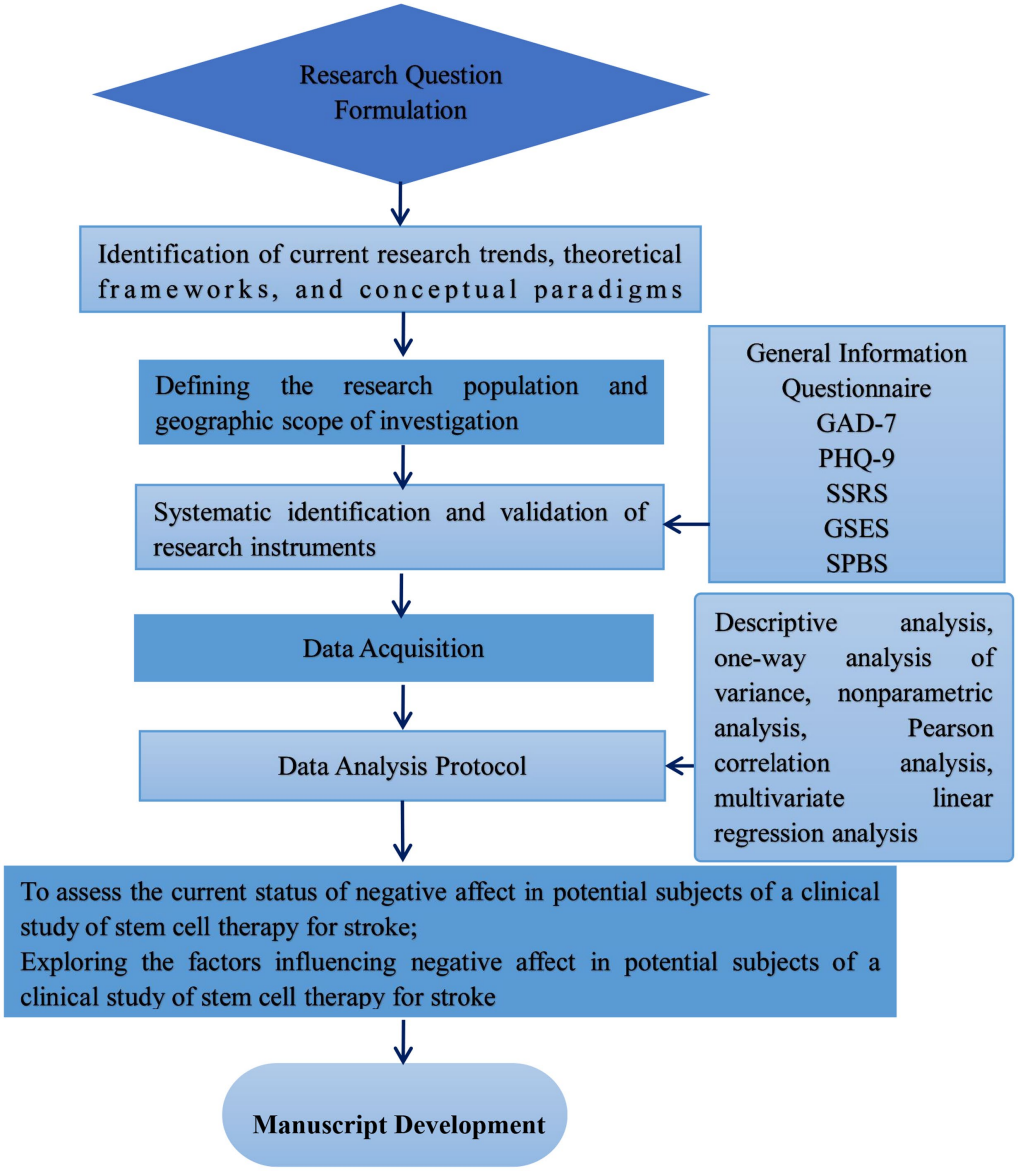
Research protocol diagram.

This study involved a multidisciplinary collaborative research team comprising experts in neurology, nursing, psychology, statistics, stem cells, and regenerative medicine. After the team members had discussed and formulated the research plan, it was submitted to the School of Nursing expert committee at Dalian Medical University for review. The expert committee reviewed and guided the revision and improvement of the standardized selection and use of scales. The revised research plan was also submitted to the expert committee for review and approval. The technical roadmap of the study protocol is shown in Figure 1.

### 2.2 Participants

Potential participants will be recruited from a large general university hospital in Liaoning, China. Patients with stroke who meet the inclusion and exclusion criteria will be identified by reviewing their case information. To ensure that meaningful and accurate data are obtained, strict adherence to inclusion and exclusion criteria is required in pretrial participant selection.

#### 2.2.1 Inclusion and exclusion criteria

The inclusion criteria are as follows: (1) Those diagnosed with ischemic stroke according to the standard diagnosis in the *Chinese Cerebrovascular Disease Diagnosis and Treatment Guidelines 2021* and brain computed tomography (or magnetic resonance imaging); (2) Aged ≥18 years old; (3) NIHSS score of 1 to 15 points; (4) patients with acute ischemic stroke within 72 h of onset; (5) the infarct area of the stroke is the internal carotid artery system, involving only one cerebral hemisphere; (6) the TOAST classification is large artery atherosclerosis or cardioembolism. (7) Patients with stroke who have learned about stem cell clinical research and are willing to participate in this study; (8) Patients who voluntarily accepted this study investigation and signed informed consent; (9) Patient have not previously used stem cells for stroke treatment.

The exclusion criteria are as follows: (1) Patients with congenital malformations of cerebral arteries and veins, including those with complete occlusion of the M1 segment of the middle cerebral artery or patients with severe disease such as cancer and stroke; (2) Patients with Parkinson’s disease, Alzheimer’s, and motor neuron disease; patients with Yuan disease, epilepsy and other neurological diseases; (3) Patients with schizophrenia, bipolar disorder, autism and other mental and psychological diseases; (4) Patients with immunodeficiency virus (HIV), syphilis, hepatitis B and C and with infectious diseases; (5) Patients with stroke during pregnancy and lactation; (6) Patients with stroke with communication disorders.

### 2.3 Pre-test

A pre-test was used to identify potential problems and improve the questionnaire. Moreover, it helped ensure the questionnaire was readable, understandable, and logical.

#### 2.3.1 Rigorous questionnaire design

Rigorous design ensures that the questionnaire is clear and that none of the included items is ambiguous or misleading. Considering the background and level of the intended respondents and using concise language and clear instructions reduces interpretation errors.

#### 2.3.2 Survey piloting

The questionnaires were distributed after the research project was introduced to potential participants who had not participated in the questionnaire design of the survey. Participation in the study was voluntary. During the pre-test, we collected the participants’ feedback and suggestions on the content and other aspects of the questionnaire.

#### 2.3.3 Data collection

We conducted in-person, face-to-face interviews with participants to gain an in-depth understanding of their views, experiences, and suggestions. Participants were closely observed, and relevant data was collected. Next, we analyzed their performance and reactions in the pre-test.

Participants were also asked to record their thoughts, feelings, and experiences in the pre-test to provide more detailed and in-depth feedback and ensure the survey was clear and comprehensible. To make the expressed content more comprehensive, clear, and accurate, some adjustments were made to the communication strategies to ensure a completion time of 20 min. The Daily Living Ability Scale scores were also extracted from the cases. The study group and expert committee discussed the modifications until a consensus was reached.

#### 2.3.4 Data collection consistency

The investigators used a unified method to conduct the questionnaires to ensure consistency and comparability of the data.

#### 2.3.5 Data verification

The data’s completeness, consistency, and accuracy were checked before analyzing them and eliminating or correcting abnormal or erroneous data. The accuracy and reliability of the questionnaire were evaluated using a test of reliability and validity. The data were collected and analyzed in a timely fashion and feedback and improvement measures were provided based on the results. This improves the questionnaire’s quality and increases the accuracy and reliability of future surveys. Implementing these quality control measures helps ensure the quality of the descriptive questionnaire and improves the reliability and accuracy of the data.

### 2.4 Questionnaire

#### 2.4.1 General information questionnaire

After reviewing the literature and based on discussions with expert members of the research team, we designed a general information questionnaire to collect data on the demographic and disease characteristics of patients with stroke. We also selected a scale that has been tested for reliability and validity in China to assess potential participants in clinical research on stem cell therapy for stroke, depression, self-efficacy, social support, and self-perceived burden.

The general patient information questionnaire includes questions regarding demographic factors, socioeconomic status, health status, and biochemical parameters. The response format is closed-ended and has the following structure: gender (female, male), age (≤ 44 years, 45–59 years, 60–74 years, 75–89 years, ≥ 90 years), marital status (married, single, widowed, divorced), education level (elementary school and below, junior high school, high school, university, graduate school), living status (living with family, nursing home, alone), place of residence (urban, rural), medical payment method (self-pay, resident medical insurance, Employee Medical Insurance), per capita monthly family income (CNY ≤ 1,000, CNY 1001–3,000, CNY 3001–5,000, CNY 5001–7,000, CNY > 7,000), sleep duration per night (≤ 4 h, 5–6 h, 7–8 h, ≥ 8 h). Questions will also include regarding the presence of other diseases (none, yes), the respondents’ ability to conduct activities of daily living (completely need help in life, need much help in life, need some help in life, basically capable of self-care), their fibrinogen level (normal, elevated, reduced), and their plasma D-Dimer level (normal, elevated, reduced).

#### 2.4.2 Additionally included scales

We will include the Generalized Anxiety Disorder 7-item scale (GAD-7) to assess the potential participants’ anxiety symptoms in the past 2 weeks. Each item ranges from 0 (never) to 3 (almost every day), with a total score of 0–21; higher scores indicate more severe anxiety symptoms (15).

The Patient Health Questionnaire (PHQ-9) comprises nine items to assess potential participants’ feelings and depressive symptoms in the past 2 weeks. Each item is scored from 0 (never) to 3 (almost every day), with the total score ranging from 0 to 27. Higher scores reflect a more serious degree of depressive symptoms (16).

The Chinese version of the General Self-Efficacy Scale (GSES-C), which has high acceptance and good reliability, is used to assess potential self-efficacy. The scale contains 10 items, and each item has four options: 1 = completely incorrect, 2 = moderately correct, 3 = mostly correct, and 4 = completely correct. Higher scores indicate better self-esteem. In addition, the scale has been shown to have high efficiency (17).

The Social Support Rate Scale (used) evaluates the social support of potential participants. The total scale score is the sum of the scores of the 10 items. The total scale score ranges from 12 to 66 points. A low level of social support is ≤22 points. The medium level ranges from 23 to 44 points, and the high level 45–66 points. The higher the total score, the higher the social support (18).

The Self-Perceived Burden Scale (SPBS) assesses the burden experienced by potential participants. The scale has 10 items. The sum of the scores for each item is the total SPBS score. The higher the score, the heavier the burden. This scale has been used to evaluate patients with stroke in China and has high acceptance and reliability (19).

### 2.5 Sample size

According to the rough estimation method for the sample size, the sample size should be at least 5–10 times the number of independent variables: the sample size N = [number of research factors (n) × (5–10 times)] (20). This study had 22 variables for statistical analysis, and the sample size collection range was 110–220 cases. To account for invalid or missing questionnaires during questionnaire recovery, the sample size was increased by 10%, and the collection range was 121–242 cases. The final sample size was set at 230.

Data collection from the planned sample of the predetermined size is expected to occur from April 2023 to April 2025 and statistical analysis will be conducted afterward. Table 1 presents the project schedule.

**Table 1:** Project timeline.

Dates	Activity
1 April 2023 to 31 July 2023	Formation of teams and training of collaborators
1 August 2023 to 31 December 2023	Visiting doctors and nurses to seek support, facilitate recruitment
1 January 2024 to 30 April 2024	Study to be modified based on pilot feedback
1 May 2024 to 31 August 2024	Study pilot
1 September 2024 to 28 February 2025	Collection of data
1 March 2025 to 30 April 2025	Analyses of data for preparation of manuscripts

### 2.6 Patient and public involvement

Posters will be displayed in the wards before and during the data collection phase to increase patient awareness of the survey. Before data collection, doctors and nurses from the relevant departments were visited to obtain their support. Based on the feedback from potential participants regarding the pre-test, appropriate modifications were made to the research design.

### 2.7 Ethics and dissemination

#### 2.7.1 Ethical approval

This study was approved by the Ethics Committee of the First Affiliated Hospital of Dalian Medical University (approval number: PJ-KS-KY-2023-124). Participant anonymity will be retained because no identifying information is included in the questionnaire. Participation is voluntary, and all participants will sign an informed consent form after fully understanding this study’s purpose, process, rights, and risks. Respondents are assigned a unique anonymous identifier after fully understanding the questionnaire content, and the researcher will strictly follow data protection laws.

## 3 Planned analyses

### 3.1 Descriptive analysis

Descriptive analysis will be conducted using the scores from the General Information Questionnaire scores, GAD-7, PHQ-9, GSES-C, SSRS, and SPBS. Results will be expressed as mean ± standard deviation and percentage. Data that do not reflect a normal distribution will be described using the median and interquartile range.

### 3.2 Statistical analysis

The collected data will be entered into an Excel database, double-checked, and subsequently imported into the SPSS 25.0 software package for data analysis. The significance level will be set to *α* = 0.05; *p* < 0.05.

#### 3.2.1 Single-factor analysis

When the scale scores and the data from the General Information Questionnaire show a normal distribution and homogeneous variances, the independent sample t-test or analysis of variance will be used. Otherwise, or if the variances are uneven, non-parametric tests will be adopted. Study participants were stratified into anatomically defined stroke subtypes based on ischemic lesion localization for subgroup-specific analyses.

#### 3.2.2 Correlation analysis

We will assess correlations between the GSES-C, SSRS, SPBS, GAD-7, and PHQ-9. Pearson’s correlation analysis will be used if the data reflected a normal distribution; otherwise, Spearman’s correlation analysis will be used.

#### 3.2.3 Multi-factor analysis of influencing factors

Multiple linear regression analysis will be conducted using the total score of patients’ negative emotions as the dependent variable (Y). Statistically significant factors and those identified in the correlation analysis will be used as independent variables (X). The model aims to analyze (1) the factors influencing the negative emotions of potential participants in clinical studies of stem cell treatment for stroke and (2) quantify the contribution of each factor to the total variation.

## 4 Discussion

This study’s main strength is that it will contribute evidence on key predictors of negative affectivity in potential participants undergoing clinical trials of stem cell therapy for stroke. The results can support stem cell clinical center researchers in intervening in potential participants’ negative emotions, which is important for clinical research managers and policymakers worldwide.

We decided to study the negative emotions of potential subjects based on our previous clinical study project on stem cell treatment of cerebral palsy in children (The study was registered in https://ClinicalTrials.gov, NCT03005249). In this study, we observed that participants who received stem cell treatment for neurological diseases had negative emotions. Stroke patients are also a group prone to negative emotions (21). We hope to understand the negative emotional characteristics of the patient group willing to receive stem cell treatment and allow us to prepare for the psychological intervention of these participants in clinical studies. Of course, the results can more broadly reflect the emotional characteristics of participants willing to participate in clinical studies and provide a reference for the development of clinical studies worldwide.

von Känel et al. (22) studied the longitudinal association between cognitive depressive symptoms and D-dimer levels in patients following acute myocardial infarction. They found that D-dimer was independently associated with depression. Patel et al. (23) found that elevated fibrinogen levels were consistently observed in patients with depression. The methodological advantage of this study is that it includes objective clinical test indicators (e.g., plasma D-dimer, fibrinogen) as independent variables, which reduced the risk of the research results being entirely caused by self-report bias. Another advantage of this study is that the assessment tool has been validated on a large scale in China, and the scale has good reliability and validity (24, 25). Epidemiological evidence shows that the incidence of stroke in young adults is on the rise worldwide (26). The incidence of stroke in adults aged 20–44 in the United States increased from 17 per 100,000 American adults in 1993 to 28 per 100,000 in 2015 (27). The incidence of stroke in young people in China is also on the rise (28). Therefore, the age standard for inclusion in this study is ≥18 years old, rather than being limited to elderly patients with stroke, which is particularly important given the trend of more strokes occurring among young people. The other advantage of this study is that the research participants are stroke patients willing to participate in stem cell clinical research, which can improve the questionnaire’s effective response and recovery rates.

This study has several limitations. First, this study is a clinical observational study, and causal relationships between the variables cannot be inferred. Second, the assessment of variables is from individuals’ self-reports with relatively few objective indicators; therefore, subjective bias may exist in the questionnaire results. Finally, including more self-report questionnaires burdens participants and can affect their patience. Additionally, several methodological limitations and unforeseen confounding effects should be acknowledged. Notably, residual confounding remains a critical concern, particularly regarding comorbidities in participants who may develop incident conditions during assessments. Inadequate adjustment for such variables could systematically bias outcome interpretations. Furthermore, emergent confounders, such as unplanned therapeutic interventions or diagnostic procedures necessitating questionnaire completion interruptions, may introduce unmeasured variability in data acquisition protocols. Thus, the quality of the questionnaire responses may be affected.

This clinical observational study will obtain the current level of negative emotions of potential participants in clinical studies on stem cell therapy for stroke and analyses the main influencing factors. This information will improve potential participants’ understanding of their negative emotions and help them obtain targeted psychological care, thereby alleviating negative emotional symptoms and promoting mental health recovery. Reducing the participant dropout rate in clinical research and improving patient engagement, compliance, retention rates, and overall satisfaction will help improve clinical research outcomes. Furthermore, the study may promote better understanding among medical staff regarding potential participants’ negative emotions in clinical research on stem cell treatment for stroke and allow scientific quantification of potential participants’ psychological care needs. This understanding is conducive to the effective allocation of nursing resources and enables nursing staff to formulate personalized care plans. In summary, this study implements the ‘bio-psycho-social-environmental’ medical model, embodying the ‘people-oriented, patient-centered’ concept and implementing high-quality care.
